# A Web-Based Well-Being and Resilience Intervention for Family Members and Friends Supporting a Loved One Using Alcohol and Other Drugs: Mixed Methods Pilot Study

**DOI:** 10.2196/72425

**Published:** 2025-07-09

**Authors:** Steph Kershaw, Jessica Deng, Madeleine Keaveny, Bronte Speirs, Anna Grager, Dara Sampson, Kate Ross, Nicola Newton, Maree Teeson, Frances Kay-Lambkin, Cath Chapman

**Affiliations:** 1 The Matilda Centre for Research in Mental Health and Substance Use, University of Sydney, 160 City Road, Level 6 G02 Jane Foss Russell Building, Sydney, 2006, Australia, 61 0286279048; 2 The University of Newcastle, Newcastle, New South Wales, Australia; 3 Hunter Medical Research Institute, Newcastle, New South Wales, Australia

**Keywords:** affected friends and family members, substance use, help-seeking, web-based intervention, pilot trial, mobile phone

## Abstract

**Background:**

Despite the known psychosocial challenges associated with supporting a loved one using alcohol and other drugs (AOD), there is a scarcity of mental health and well-being interventions for affected friends and family members (AFFMs). Stigma has also been shown to discourage help-seeking among AFFMs. Web-based interventions may facilitate help-seeking by ensuring privacy and anonymity.

**Objective:**

This pilot study examines the usability, acceptability, and feasibility of the Family and Friend Support Program (FFSP), a world-first, evidence-based web-based resilience and well-being program designed with, and for, people caring for someone using AOD. This study also examined AFFM’s experiences of caring for a loved one using AOD and their help-seeking behaviors and barriers.

**Methods:**

In 2021 (November-December), participants across Australia completed a baseline web-based cross-sectional survey that assessed the impact of caring for a loved one using AOD (adapted Short Questionnaire for Family Members-Affected by Addiction), and distress levels (Kessler Psychological Distress Scale [K-10]). Following baseline, participants were invited to interact with the FFSP over 10 weeks. Postprogram and follow-up surveys (10 and 14 wk postbaseline, respectively) and semistructured interviews assessed the usability and acceptability of the program, as well as help-seeking experiences and barriers.

**Results:**

Baseline surveys were completed by 131 AFFMs, with 37% (n=49) completing the postprogram survey and 24% (n=32) completing the follow-up survey. A total of 5 participants took part in individual semistructured interviews at postprogram. On average, K-10 scores fell in the moderate to severe range at baseline (mean 28.4, SD 8.6). At postprogram, the majority of participants (n=27, 55.1%) reported that they did not seek help to cope with or manage their role supporting their loved one and the most common endorsed barrier was cost (n=11, 28.6%). Overall, participants found the FFSP easy to use and provided them with relevant, helpful, and validating information. The majority (n=35, 71.5%) of participants said they would be likely to recommend the FFSP to a person supporting a loved one using AOD. Qualitative responses highlighted the need for free, accessible support for AFFMs such as the FFSP. Limitations included low program engagement and high attrition.

**Conclusions:**

Overall, the FFSP appears to be a promising mental health intervention for AFFMs. This study builds on existing research finding high levels of distress among AFFMs, while highlighting the ongoing barriers to help-seeking. Limitations and future directions for refinements and future efficacy evaluation of the FFSP are discussed including ways to address attrition and increase engagement.

## Introduction

The use of alcohol and other drugs (AOD) not only affects the person using the substance but also impacts their family, friends, and community, often in adverse ways [1,2]. The 2022-2023 National Drug Strategy Household Survey found that around 1 in 3 Australians (31% or 6.6 million) reported drinking alcohol above guideline recommendations [3], and around 1 in 5 (18% or 3.9 million) people had used an illicit drug in the previous 12 months [4]. Additionally, the Australian Burden of Disease Study reported that AOD use was estimated to be responsible for 7.5% of the total burden of disease and injury [5]. Considering the high rates of AOD use and associated burden in Australia, the number of family members and friends who are supporting someone using AOD, or are affected by someone’s use, is likely to be high [6].

The term “affected friends and family members” (AFFMs), used in this paper, represents a heterogeneous group that includes parents, children, significant others, relatives, friends, and caregivers of someone using AOD. AFFMs often have the responsibility of advocating and caring for their loved one, while also managing their loved one’s changes in behavior, increased relationship strain and conflict, and financial insecurity [1,7-9]. Taking on this support role in these circumstances can be difficult and unpredictable and it has been found that AFFMs experience a substantially poorer quality of life compared to the general population, including poorer physical and mental health [10-13]. AFFMs may be at higher risk of developing AOD use, depression, and anxiety disorders themselves [6,14]. As AOD use and dependence can be a lifelong issue, there is a need to support AFFMs to develop effective long-term coping strategies and access support for their own needs and well-being [15].

AFFMs also face significant stigma and social isolation, which can further exacerbate stress and their ability to cope and seek support [16,17]. As well as public and self-stigma associated with substance use, AFFMs may be subject to stigma by association or “courtesy stigma” which involves public disapproval and negative social interactions invoked by narratives of blame, shame, and contamination around those supporting a loved one using AOD [18,19]. Both public stigma and stigma by association have been found to be a significant barrier to help-seeking. AFFMs may be deterred from seeking help due to shame, fear of judgment, and concerns around privacy for both themselves and their loved ones [20]. However, help-seeking, both informally and formally, has been identified as an important strategy for helping AFFMs manage and cope adaptively with stressors and reduce levels of impact and burden [7,21,22]. Thus, addressing barriers of stigma and shame associated with seeking help is critical when developing and promoting effective forms of support and interventions for AFFMs.

Previous research suggests that the experiences and impact of supporting a loved one may depend on characteristics such as the AFFM’s gender and relationship with their loved one, as well as on sociocultural differences [23-25]. For example, female partners, parents, and those who live in the same household as their loved one are more likely to experience a greater cumulative burden compared to other family members and friends [26,27]. Differences have also been found based on country and geographical location, suggesting cultural variations in impact and coping among AFFMs [23,24,28,29]. Therefore, it is important to consider potential differences in impact and coping based on demographic factors to appropriately capture the different needs of this diverse population.

Research now conceptualizes AFFMs as an independent population who are uniquely impacted by the significant uncertainty, stress, and strain that may arise around a loved one’s AOD use. Historically, AFFMs have been seen as “part of the problem” or only as an adjunct to treatment of the individual affected by AOD use rather than as help-seekers in and of themselves [16,30,31]. In response to this, Orford et al [32] developed the Stress-Strain-Coping-Support Model which is a nonpathological model that recognizes the chronic stress of living with or supporting an individual experiencing drug dependence and the way this stress leads to strain which is mediated by their coping style and quality of social support. The 5-step method puts this model into practice with an emphasis on empowering family members, reducing distress, providing information, and enhancing coping and support [33]. This model has been shown to be effective in reducing the strain experienced by family members with some positive knock-on effects for the person they are supporting [15]. This finding is consistent with previous research suggesting that family members and family function play a key role in preventing and minimizing the risks and harms associated with a loved one’s AOD use, as well as promoting resilience and more positive and longer-lasting treatment outcomes [30,34-36]. Specifically, family-focused approaches to intervention and recovery improve family functioning, reduce relapse, and help both affected family and their loved ones improve their quality of life [35]. This signifies the importance of fostering help-seeking among AFFMs, and the need for effective, targeted interventions for this population.

To date, there are few interventions available to AFFMs that do not require the involvement of the person using AOD, and the interventions that do exist are rarely evaluated [7]. Two recent web-based group programs developed specifically for AFFMs have shown promising results in terms of self-reported outcomes and feasibility [37,38]. The peer-led web-based support program by Peart et al [38] found significant improvements in self-efficacy and overall satisfaction with the program, and Rushton et al [37] found that the SMART Family and Friends, a mutual-support group targeting families delivered via videoconferencing, was associated with significant improvements in psychological distress, family impact, strain symptoms, and total family burden. These programs demonstrate the need for support and interventions specifically developed to address the unique challenges faced by AFFMs, such as web-based groups and peer-based support.

While web-based peer support groups offer a valuable avenue for accessing help, it is important to consider the ongoing barriers to help-seeking including stigma, gaps in help-seeking knowledge, concern for their own and their loved one’s privacy, and practical concerns (eg, cost, location, and time) which can deter AFFMs from accessing support even when it exists [17,39,40]. Additionally, AFFMs often face multiple competing stressors on top of and related to caring for a loved one using AOD and this can lead to carer burnout, introducing an additional barrier to participating in structured, group-based interventions [41,42]. These barriers and the isolating impacts of stigma highlight the need for interventions to be privately accessible anywhere at any time for AFFMs.

To address this gap, the Family and Friend Support Program (FFSP) [43] was developed in 2019 [44]. The FFSP is a world-first, evidence-based, web-based intervention designed with, and for, family members and friends supporting loved ones using AOD. The development of the FFSP was based on the Stress-Strain-Coping-Support Model and the 5-step method [15] and included consultation and collaboration with AFFMs, capturing their concerns and needs through web-based surveys and interviews. The program provides tailored support for AFFMs that is free, confidential, and accessible digitally anywhere at any time across Australia. This allows vulnerable and burdened AFFMs to access support without anxieties around compromising their own or their loved one’s privacy. It also ensures that at-risk groups and marginalized communities have access to the program including women (who often bear the greater caregiver burden), low socioeconomic communities (who experience greater financial stress), and those living in rural, regional, or remote areas with limited resources [1,39,45]. In this way, the FFSP is positioned as a low-barrier early-intervention option for AFFMs learning to manage their role as a carer and advocate for their loved ones using AOD. The FFSP features a package of modules informed by principles of cognitive behavioral therapy including psychoeducation, personalized activities, and information on other web-based resources and services [44]. With permission, the FFSP also included excerpts from initial AFFM consultations and interviews conducted during the development of FSSP as quotes and vignettes throughout the program to highlight lived experience perspectives (see Multimedia Appendix 1 for program screenshots).

This pilot study aimed to assess the usability, acceptability, and feasibility of FFSP. A secondary aim was to capture a cross-sectional snapshot of a diverse sample of AFFM’s experiences of caring for a loved one using AOD.

## Methods

### Study Design

The study involved web-based surveys completed at three timepoints: (1) baseline, (2) postprogram (10 wk postbaseline), and (3) one-month follow-up (14 wk postbaseline; see Multimedia Appendix 2 for study flow). The baseline survey assessed demographic details about participants and their loved ones, psychological distress, and family burden including impact, stress, coping, and social support. Following baseline surveys, participants were invited to interact with the FFSP [43] and complete 11 evidence-informed modules, including 4 core modules and 7 mini modules, over a 10-week period. The postprogram survey was offered at 10 weeks postbaseline to all AFFMs who completed baseline, regardless of whether they had accessed all, part, or none of the FFSP. The same sample was offered a follow-up survey 4 weeks later. The postprogram and follow-up surveys assessed previous help-seeking experiences and barriers, program usability and feedback, and repeated baseline measures of psychological distress and family burden. Participants were offered the opportunity to volunteer for an in-depth phone interview at both postprogram and follow-up timepoints.

### Participants and Recruitment

Between November and December 2021, Australian residents from the general community aged 18 years or older were recruited for the web-based survey via social media (Facebook, Twitter) along with e-newsletters. Recruitment advertisements targeted AFFMS (eg, “Have you been affected by someone else’s use of drugs or alcohol? Researchers at the University of Newcastle and the University of Sydney have developed a web-based program and information website to help families or friends”). All 3 surveys were administered via REDCap (Research Electronic Data Capture; Vanderbilt University) [46,47], a secure, web-based data capture tool. Semistructured phone interviews were conducted via Zoom (Zoom Video Communications Inc) or telephone depending on the participants’ preference with participants confirming at the start of the interview that they were in a safe, private space (eg, a room in their own house). Inclusion criteria were being an Australian resident, older than 18 years of age, and having a close family member or friend whose AOD use was causing them concern. Since this was a pilot study, a sample size calculation was not performed. The researchers aimed for 120 participants as it was believed this would be a large enough sample to gain insight into the usability, acceptability, and feasibility of the web-based program accounting for a moderate level of attrition.

### Measures

#### Demographics

Demographic information included age (in years), gender (“How do you identify?”), residential postcode, geographic region (metropolitan, regional, or rural or remote), and cultural heritage [48]. Participants were also asked how they typically accessed the internet (smartphone, PC, or tablet), and questions about their family member or friend who uses AOD including demographics, the substance of concern, length of use, and the participant’s relationship to that person (eg, partner, friend).

#### Psychological Distress

Psychological distress was measured using the Kessler Psychological Distress Scale (K-10) [49]. The K-10 is a well-validated and widely recommended simple screening measure of psychological distress [50]. Participants responded to 10 items about their emotional states in the past 4 weeks on a 5-point Likert scale (where 1=none of the time and 5=all of the time). Scores were then summed with the maximum score of 50 indicating severe distress, and the minimum score of 10 indicating no distress.

#### Impact, Stress, Coping, and Social Support

Stress, coping, and social support were measured via the adapted Short Questionnaire for Family Members-Affected by Addiction (SQFM-AA) [51]. The SQFM-AA has shown satisfactory to good internal reliability and validity [27]. The SQFM-AA consists of 33 questions about a loved one’s AOD-related behaviors in the preceding 3 months and participants rated how often they have engaged in certain responses using a 4-point Likert scale (1=never, 2=once or twice, 3=sometimes, and 4=often). The SQFM-AA includes subscales measuring impact (worry and disturbance), symptoms (physical and psychological), coping (engaged emotional, engaged assertive, tolerant, and withdrawal), social support (helpful formal, helpful informal, and unhelpful informal), and total family burden. Total family burden scores include the cumulative score of impact, symptoms, and coping with scores ranging from 0 to 48 and higher scores indicating a higher degree of burden experienced by the family member.

#### Help-Seeking Experiences

At postprogram and follow-up, participants were also asked about their previous help-seeking experiences: “Over the past month, did you seek help to help cope with or manage your role supporting your loved one (eg, mental health support)? Yes or No” and if they answered “Yes,” they were prompted to select all that applied to them from a list of services including “Online,” “GP,” “Counsellor or psychologist,” and “Friend or family member.” Participants were then asked, “Did you receive the help that you needed?” (Yes or No), followed by “Please comment on the help you did or did not receive” (free textbox). All participants were asked about barriers to help-seeking. Barriers to help-seeking were assessed using the barriers to help-seeking scale, adapted from the widely used, reliable, and valid Perceived Need for Care Questionnaire administered in the Australian National Survey of Mental Health and Well-being [52,53]. Participants were asked “Were there any barriers that have stopped you from seeking help (Select all that apply)?” and then asked to select from 11 predefined statements. Additionally, there was an option to select “Other” followed by the prompt “Please specify other barriers that have stopped you from seeking help?” and a free textbox.

#### Usability, Acceptability, and Perceived Usefulness of the Program

Program usability was assessed via the System Usability Scale (SUS) [54]. The SUS reliably measures usability and consists of 10 items including several facets such as ease of website use and website complexity. These 10 items were scored on a 5-point Likert scale ranging from 0=strongly disagree to 5=strongly agree. Higher scores indicate better usability and range from 0 to 100. Scores of 80 or above indicate a strong performance, with the average SUS score being 68 as based on more than 500 studies [54].

Additionally, participants answered the Likert scale (eg, “How easy was it to find the information you wanted? (Select an option: Very easy, moderately easy, somewhat easy, not very easy, not easy at all, unsure)”) and open-ended questions (eg, “Was there anything missing from FFSP that you expected or wanted to be included?”) about the acceptability and usefulness of the FFSP in terms of structure and content. Participants also answered open-ended questions about perceived barriers to accessing the program (“What barriers stopped you from accessing FFSP (specify)?”), likelihood of recommending to someone else (“On a scale of 1 (would not recommend at all) to 10 (extremely likely to recommend), how likely would you be to recommend FFSP to a person supporting a loved one using alcohol or other drugs?”), and feedback on ways to improve the program (“Was there anything missing from FFSP that you expected or wanted to be included? If yes, then, what was missing (specify)”).

#### In-Depth Phone Interviews

Further, optional in-depth semistructured phone interviews were conducted by a clinical psychologist or social worker experienced in interviewing. Confidentiality was explained to the interviewee before obtaining consent to audio record the interview which was later transcribed by a member of the research team.

### Data Analysis

Data were analyzed using the SPSS Statistics (version 25; IBM Corp). Independent samples 2-tailed *t* tests and one-way ANOVAs were conducted to assess differences in baseline K-10 and SQFM-AA Total Family Burden scores based on demographic factors (age, gender, geographical location, and relationship to loved one using AOD). To assess attrition, independent samples 2-tailed *t* tests were conducted to compare participants who completed only baseline versus both baseline and postprogram, as well as participants who accessed versus did not access the program at postprogram. Although analysis of changes in outcomes across timepoints was intended, it was not conducted due to the small sample size (n=11) of those who reported accessing the program and completed all 3 timepoints. This paper therefore reports findings from the quantitative data collected at baseline, as well as the qualitative data from postprogram and follow up.

### Ethical Considerations

Ethics approval was obtained from the University of Newcastle (H-2017‐0040) Human Research Ethics Committee. All participants provided informed consent. All data collected from participants were anonymous and nonidentifiable via the secure REDCap survey platform. Participants who completed the postprogram and follow-up surveys were reimbursed with AUD $50 (US $32) grocery digital gift cards for each survey. Participants who completed the interview were reimbursed with an AUD $25 (US $16) grocery digital gift card.

## Results

### Demographics and Sample Characteristics

A total of 131 participants completed baseline measures (refer to Table 1 for characteristics). Mean age was 50.9 (SD 12.7) years and 80.2% (n=105) identified as female. Just over half of the participants were from metropolitan areas (n=68, 51.9%) with the majority born in Australia (n=107, 81.7%). The most common relationship of the loved one was a child (n=50, 38.2%), and the most reported substance of concern was ice or crystal methamphetamine (n=64, 48.9%).

**Figure 1.**
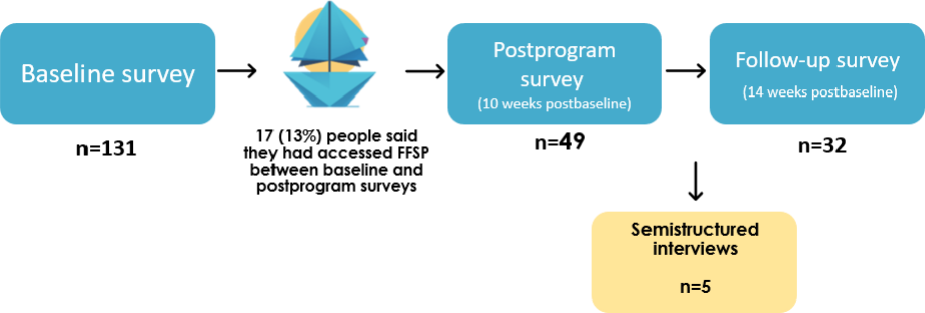
Flowchart of the study design for affected friends and family members (n=131 at baseline), which includes the sample sizes at each survey timepoint (baseline, postprogram, and follow-up), whether they accessed the FFSP, and participation in optional in-depth interviews. FFSP: Family and Friend Support Program.

A total of 49 (37%) participants went on to complete the postprogram survey and 32 (24%) participants completed the follow-up survey. At the postprogram timepoint, 17 (13% of the baseline sample) participants reported accessing the FFSP, while at the follow-up survey, 14 (11% of the baseline sample) participants had accessed the FFSP since postprogram. A total of 5 (4%) participants took part in individual semistructured interviews. Figure 1 shows the study flowchart.

**Table 1.** Demographic characteristics of the affected friends and family members sample at baseline survey.

Characteristics	Value (n=131)
Age (years), mean (SD)	50.9 (12.7)
Gender, n (%)	
Woman	105 (80.15)
Man	25 (19.1)
Nonbinary	1 (0.8)
Country of birth, n (%)	
Australia	107 (81.7)
Other^a^	24 (18.3)
Geographical area, n (%)	
Metropolitan	68 (51.9)
Regional	42 (32.1)
Rural or remote	21 (16)
Loved one using AOD^b^, n (%)	
Child^c^	50 (38.2)
Partner^d^	29 (22.1)
Sibling	11 (8.4)
Friend	10 (7.6)
Parent	10 (7.6)
Other^e^	21 (16)
AOD of concern, n (%)	
Crystal methamphetamine	64 (48.9)
Alcohol	40 (30.5)
Cannabis	12 (9.2)
Heroin	4 (3.1)
Prescription opiates	4 (3.1)
Other	7 (5.4)

a Other includes Afghanistan, Canada, Indonesia, Iran, Malaysia, Netherlands, New Zealand, Scotland, Singapore, South Africa, United Kingdom, and the United States.

b AOD: alcohol and other drugs.

c Child includes son, daughter, step-son, and step-daughter.

d Partner includes husband, wife, spouse, and partner.

e Other includes ex-partner, sibling-in-law, nephew, roommate, and neighbor.

### Analysis of Dropout and Attrition

As attrition (62.6% dropout) between baseline and postprogram timepoint was high, analysis was undertaken to explore if there were any demographic differences between those who completed only the baseline survey and those who completed both the baseline and postprogram survey. Age, gender, geographical location, and relationship to the affected loved one were not significantly associated with dropout from baseline to postprogram. There were also no significant differences in basic demographic factors between those who did and did not access the program.

### Psychological Distress at Baseline

The mean K-10 score at baseline among the total sample was 28.4 (SD 8.6), which falls in the moderate to severe range of psychological distress (Multimedia Appendix 3). There were no significant differences in K-10 scores based on age, gender, relationship to affected loved one, or geographical location. However, participants who accessed the FFSP had significantly higher baseline K-10 scores (mean 31.6, SD 10.3) compared to those who did not access the FFSP (mean 24.2, SD 7.6; *t*_47_=2.86; *P*=.006).

### Stress, Coping, and Social Support

#### Stress, Strain, and Total Family Burden

The mean total family burden score at baseline was 44.96 (SD 9.46), indicating a high degree of burden experienced by participants associated with their loved one’s AOD use. Total family burden did not differ significantly based on demographics such as age, gender, relationship to loved one, or geographical location.

In terms of the impact of their loved one’s AOD use over the previous 3 months, the majority of participants reported that their loved one’s AOD use at least sometimes affected the family’s finances (n=89, 68%), got in the way of their social life (n=99, 75.6%), led to worry about their loved one’s neglect of self-care (n=104, 79.4%), led to fights or arguments (n=96, 73.3%), or upset family or social occasions (n=81, 61.9%). More than half reported that their loved one has threatened them at least once or twice (n=71, 54.2%). The majority (n=93, 71%) of participants reported often worrying about their loved one’s AOD use. Almost half the participants reported sometimes having difficulty concentrating (n=77, 48.9%) and feeling weak in parts of their body (n=64, 48.9%).

#### Coping Styles

One-third (n=39, 29.8%) of participants endorsed often watching their loved one’s every move, 15% (n=19) had often started an argument with their loved ones about their AOD use, and 30% (n=37) often got moody and emotional with their loved ones. One-third of participants endorsed often talking frankly about their AOD use and making clear to their loved ones their expectations of how they should contribute to the family. Over a third (n=45, 35%) endorsed often putting themselves out for their loved ones, for example, getting them to bed or clearing up the mess after they had been drinking or taking drugs.

#### Social Support

A majority of participants (n=88, 64.1%) endorsed that friends or relations sometimes or often listened to them when they talked about their feelings, that their friends or relations sometimes or often have been there for them (n=83, 63.3%), and that friends or relations have sometimes or often talked to them about their loved one and listened to what they had to say (n=82, 62.6%). Over half the participants endorsed that they never had health or social care workers give them helpful information about problem drinking or drug taking (n=76, 58%), had health or social care workers make themselves available to them (n=77, 58.8%), or confided in their health or social care worker about their situation (n=70, 53.4%; see Multimedia Appendix 4 for a full breakdown of SQFM-AA scores).

### Help-Seeking Experiences

Among the participants who completed the postprogram survey (n=49), the majority (n=27, 55.1%) reported that they did not seek help to cope with or manage their role in supporting their loved ones. Of the 22 (44.9%) participants who reported that they sought help, the most common form of support accessed was a counselor or psychologist (n=13, 26.5%). Participants provided a mix of positive and negative comments on their previous help-seeking experiences. Examples of positive comments include, “My psychologist has been a great listener and has been very understanding and supportive” and “NA (Narcotics Anonymous) has been useful in that we now have a small group of people who understand our situation and are supportive. Otherwise, NA and 12 steps in particular don’t feel relevant or useful to me.” Examples of negative comments include, “(health workers’) Judgment or jumping to conclusions” and “too long of waitlists.” These sentiments were also reflected in interviews, for example, one interviewee highlighted that stigma is an ongoing barrier to asking for help.

*One barrier that I think I could identify there is that it’s very common, in my experience, that people, family and friends of people who’ve got a substance abuse problem ... feel almost embarrassed to admit that this is something that’s completely out of their control, and participating in programs can often be a very challenging thing for them to do, because they fear that they’re going to be held accountable.*

Barriers to help-seeking are summarized in Table 2.

**Table 2.** Barriers to help-seeking endorsed by affected friends and family members at postprogram survey completed 10 weeks postbaseline^a^.

Barrier to help-seeking	Value (n=49), n (%)	
I couldn’t afford the money	14 (28.6)	
I didn’t need help	11 (22.4)	
I preferred to manage myself	11 (22.4)	
I didn’t think anything could help	9 (18.4)	
I was afraid of legal implications	9 (18.4)	
I previously asked but didn’t get help	9 (18.4)	
I couldn’t afford the time off work	8 (16.3)	
I previously asked for help but had a negative or bad experience	6 (12.2)	
I was afraid of what others would think of me	4 (8.2)	
I didn’t know where to seek help	4 (8.2)	
I was afraid to seek help	2 (4.1)	
Other^b^ (service unhelpful, not needing support, general stigma, other support)	6 (12.2)	

a Note that participants were able to select multiple responses.

b Free textbox.

### Usability, Acceptability, and Perceived Usefulness of the Program

The average SUS score for the FFSP was 70 (SD 18; n=17), which is slightly above the average score of 68 from 500 studies [54]. Among the participants who completed the postprogram survey, the majority (n=36, 65.3%) of participants reported that it was easy to find the information that they wanted, and most (n=35, 71.4%) reported that the information was easy to understand. Further, over half (n=26, 53.1%) felt that the activities were somewhat, moderately, or very helpful. After interacting with the FFSP, 1 in 3 (n=17, 34.7%) felt very or moderately confident when faced with AOD-related issues. The majority (n=35, 71.5%) of participants rated that they would be likely to recommend the FFSP to a person supporting a loved one using alcohol or other drugs.

All interviewees in the semistructured interviews (n=5) identified the need for programs such as the FFSP to address the lack of accessible support for AFFMs, to validate and normalize AFFMs experiences, and to combat misinformation and stigma surrounding AOD use. For example, one interviewee found the vignettes of lived experience stories validating.

*It’s extremely comforting... to find that there is somebody else who is just like you*.

Another interviewee spoke about the important role of web-based support in overcoming shame.

*There is a big role for online support to play because it helps people not feel so bad because I think ... half the problem is, people don’t tell anyone because it’s so shameful, particularly once they’re an adult*.

Another interviewee highlighted the need for web-based, 24/7 access particularly when living in a small town*.*

*Being in a smaller town where everyone seems to know everyone’s business ... makes it a bit harder to front up to those sort of things [ask for help]. So when you can do stuff online it can be obviously done in your own time*.

Some interviewees described the length and amount of content as a barrier to completion. For example, one participant found that “FFSP felt quite complex and needed considerable time commitment” and another participant noted that *“*there was just so much information, it required considerable time to work through*.*” In terms of ways to improve the content of the FFSP, participants suggested including more practical strategies and skills for supporting their loved ones, more content focused on lived experiences, and follow-up with in-person community groups and resources. For example, when asked what they would like to include, one interviewee said, “more stories of what being a friend or family support person might involve*.”* Another interviewee wanted more content addressing stigma and shame in the carer role.

*I think it would be very helpful if you could give to people going through the program, tips to enable them (a) to understand this is not your fault, (b) to come to the realization that you don’t have to be angry at the person who’s in substance abuse.*

Finally, another interviewee said that they would like the web-based format to be complemented by in-person services and resources.

*It needs direct references into live communities of belonging, not just web resources.*

Reasons for not accessing the program between the survey timepoints included time restrictions, other stressors (eg, their own sickness, work stress, and carer burnout), and perceived lack of need (eg, receiving other formal or informal support and loved ones doing well or in recovery). Several participants also noted technical difficulties (eg, links not working and forgetting passwords).

## Discussion

### Principal Findings

This pilot study investigated the usability, acceptability, and feasibility of the FFSP, a world-first, evidence-based web-based intervention developed to support the well-being of family members and friends with a loved one using AOD.

Overall, the FFSP demonstrated good usability and acceptability among AFFMs and was found to be useful in validating users’ experiences and addressing stigma around seeking help. Most participants reported that the FFSP was easy to use, and it was easy to find and understand the content. Participants reported that the lived experience vignettes on the FFSP were validating and normalized their own experiences. This feedback is reflective of broader research highlighting the value of sharing lived experiences to reduce stigma and discrimination around mental health and substance use for both individuals and the general public [55-57]. Similar to peer support programs, the sharing of lived experience stories plays an important role in empowering AFFMs by validating their unique difficulties supporting a loved one with AOD use while also normalizing seeking and accessing support for themselves [58]. Participants highlighted that they would like to see even more emphasis on stories and voices of lived experience in the FFSP.

The findings indicate that the FFSP was feasible to administer as a web-based, self-paced program that users are able to access at their discretion. The private and confidential format helps to address participant’s concerns about stigma and fear of judgment. Further, the program being free, self-paced, and available 24/7 helps overcome other help-seeking barriers that participants endorsed such as financial strain, time restrictions, and other life stressors.

This study also examined AFFM’s experiences of caring for a loved one using AOD in relation to their levels of distress, as well as their experiences of stress and strain, coping, and social support. On average, AFFMs in this study reported moderate to severe levels of psychological distress. This is in line with previous research highlighting AFFMs as a population may be vulnerable to increased risks of psychological distress and mental health difficulties [2,13,14]. Distress levels were similar across demographic variables including age, gender, geographical location (metropolitan, regional, or remote areas), and relationship to their loved ones. Similarly, there were no significant differences in SQFM-AA scores across these participant characteristics. For example, scores measuring the degree of burden falling on an AFFM due to the effects of their loved one’s AOD use did not differ based on their relationship with the loved one. This finding is interesting as previous studies have found significant differences in symptoms of stress and level of harm based on gender, cultural factors, and relationship to the family member [26]. For example, a previous study in Brazil found that wives and mothers reported higher levels of burden compared to other family members including fathers [26]. A smaller sample size in this study, as well as uneven distribution across types of relationships and cultural backgrounds, may partially explain this different finding. Although this study did not focus on this, it would be important for future studies to explore the differences between social and cultural factors in order to inform the development of services that are culturally sensitive and responsive [59,60]. Finally, the majority of participants did not access or receive helpful support from formal services, consistent with previous findings of low rates of help-seeking among AFFMs which may also reflect a lack of accessible or appropriate formal support available [17].

Finally, higher levels of psychological distress were significantly associated with accessing the program. This is in line with this study’s finding that the most common reason for participants not accessing the program was a perceived lack of need. Almost one-third of the reasons given for not accessing the program were related to the participant’s loved one being in recovery or generally doing well. This may reflect the fact that recovery is a lifelong dynamic process dependent on individual, social, and contextual factors [61-64]. Knowing that AFFMs are greatly impacted by their loved one’s AOD use, AFFMs’ own mental health and support needs are also likely to change over time depending on both their loved one and their own life events. This points to the importance of ensuring programs such as the FFSP are a known option for AFFMs to access when they need it. This might include liaising with clinicians and health workers who are likely to be in contact with people during periods of difficulty who may then be able to refer individuals to the FFSP, positioning the program as a complementary tool in their broader recovery journey.

### Strengths, Limitations, and Future Directions

A key limitation of this study was the low rate of program uptake and high attrition, which hindered the study’s aim to assess the program’s effectiveness, as well as limited the generalizability of the findings. As feedback on usability and acceptability was drawn from a small sample, these should be interpreted as preliminary, and future studies with larger, more representative samples are needed to build upon these findings. Low uptake of the program may be due to the recruitment strategy being focused on completing the surveys rather than as an opportunity to use and test the program itself. Future research recruitment could benefit from clearly communicating the commitment required in signing up for the study, reimbursing participants at baseline to incentivize return, and increasing email reminders and contact with the research team between surveys to troubleshoot technical barriers. Further, the reliance on web-based recruitment platforms may have also biased our sample toward more educated and digitally literate populations. Future research recruitment could benefit from partnering with key AOD organizations (eg, Family Drug Support) or posting in AOD community forums to help reach a more diverse group of potential participants. High attrition may also be partly due to the impact of the pandemic and natural disasters occurring over the course of recruitment, however, poor adherence to web-based mental health and substance use interventions is a universal problem [65,66]. Further, AFFMs are a particularly vulnerable and hard-to-reach population facing ongoing barriers of stigma and carer burnout [17]. It is possible that some participants simply were not at a place in their journey where they felt able and ready to participate in the program or that they needed any additional support.

Previous research has found that integrating therapist or facilitator elements into web-based interventions tends to be associated with higher rates of engagement compared to self-guided interventions [67,68]. Thus, future strategies to promote engagement and retention of the FFSP might include nonclinician facilitation such as periodic email or text reminders, technical support, “gamification” (more structured or personalized progress tracking), peer-support elements (eg, moderated discussions), or clinician guidance such as complementing brief psychosocial support from a trained therapist. Further, enhancing usability and accessibility through optimizing user experience, particularly for mobile may increase engagement and adherence as most people access the internet on their smartphone [69]. To address technical difficulties encountered by participants (eg, broken links or forgetting passwords), it would be valuable to incorporate and evaluate user-friendly enhancements such as a digital skills guide or tutorial embedded into the program to help AFFMs navigate and benefit from the program. In addition, regular reviews of the program and provision of digital technical support (eg, through the chat function) would help to quickly identify and resolve issues. Finally, future content improvements could include more lived experience stories, prioritizing clarity and conciseness of information per page, and additional resources and links to in-person services to provide follow-up and encourage people to build a network of support options. These learnings will guide a future large-scale trial evaluating the efficacy and outcomes of the FFSP.

This study also had a number of strengths. First, an intention-to-treat approach was followed which aimed to minimize the risk of bias related to adherence and attrition in regard to assessing the acceptability of the FFSP. This allowed us to capture more diverse perspectives and account for potential differences between those who do and do not access the program and those who do and do not complete the postprogram and follow-up surveys.

Second, as reflected in the broader literature, AFFMs are a particularly hard-to-reach population in research and health care considering the unpredictability of their carer roles and the concurrent burdens they face, as well as the ongoing impacts of stigma and social isolation. This study was successful in recruiting a relatively representative sample of AFFMs which included a range of genders, ages, geographical locations, and relationships to the loved one using AOD. The flexibility of the FFSP allows family members and friends to access the program 24/7 when they have the time, privacy, and space to access support. This flexibility is key to reaching at-risk groups including women (who often bear the greater caregiver burden), low socioeconomic communities (who experience greater financial stress), and those living in rural, or regional, or remote areas with limited resources [1,39,45]. Further, a number of participants reported a perceived lack of need for treatment at the time of completing the study which reflects the nonlinear nature of AOD recovery and fluctuating need for support. This points to the importance of continuing to make the FFSP readily available so that AFFMs can access the program when and wherever they may need it. As reflected in the quantitative and qualitative feedback on the program, participants provided positive feedback on the ease, convenience, and need for web-based programs.

### Conclusions

This study adds to the growing body of literature demonstrating high levels of distress and strain among people caring for a loved one using AOD, while highlighting the ongoing barriers to help-seeking among this population, as well as the opportunities for early intervention and support. This pilot study found that the FFSP is feasible to administer, acceptable, and has the potential to fill an important gap in services specific to family and friends supporting a loved one using AOD. This study also identified areas for improvement of the program and highlighted important learnings prior to conducting an evaluation of program efficacy. While the FFSP is a promising intervention for AFFMs, there is an ongoing need for increased research and investment into reducing stigma and other barriers to help-seeking, as well as developing and evaluating early intervention and support for both people who use AOD and the family members and friends who care for them.

## Supplementary material

10.2196/72425 Multimedia Appendix 1 Screenshots from the program.

10.2196/72425 Multimedia Appendix 2 Study procedure.

10.2196/72425 Multimedia Appendix 3 SQFM-AA (Short Questionnaire for Family Members-Affected by Addiction) individual item endorsement.

10.2196/72425 Multimedia Appendix 4 K-10 (Kessler Psychological Distress Scale) and SQFM-AA (Short Questionnaire for Family Members-Affected by Addiction) descriptive statistics.

## Abbreviations

AFFM: affected friends and family members
AOD: alcohol and other drugs
FFSP: Family and Friend Support Program
K-10: Kessler Psychological Distress Scale
REDCap: Research Electronic Data Capture
SQFM-AA: Short Questionnaire for Family Members-Affected by Addiction
SUS: System Usability Scale
