# Supplementary figures and images for: A Web-Based Well-Being and Resilience Intervention for Family Members and Friends Supporting a Loved One Using Alcohol and Other Drugs: Mixed Methods Pilot Study
Source: JMIR Form Res. 2025 Jul 9;9:e72425. doi: 10.2196/72425 (PMC12266297; doi:10.2196/72425)

FFSP landing page


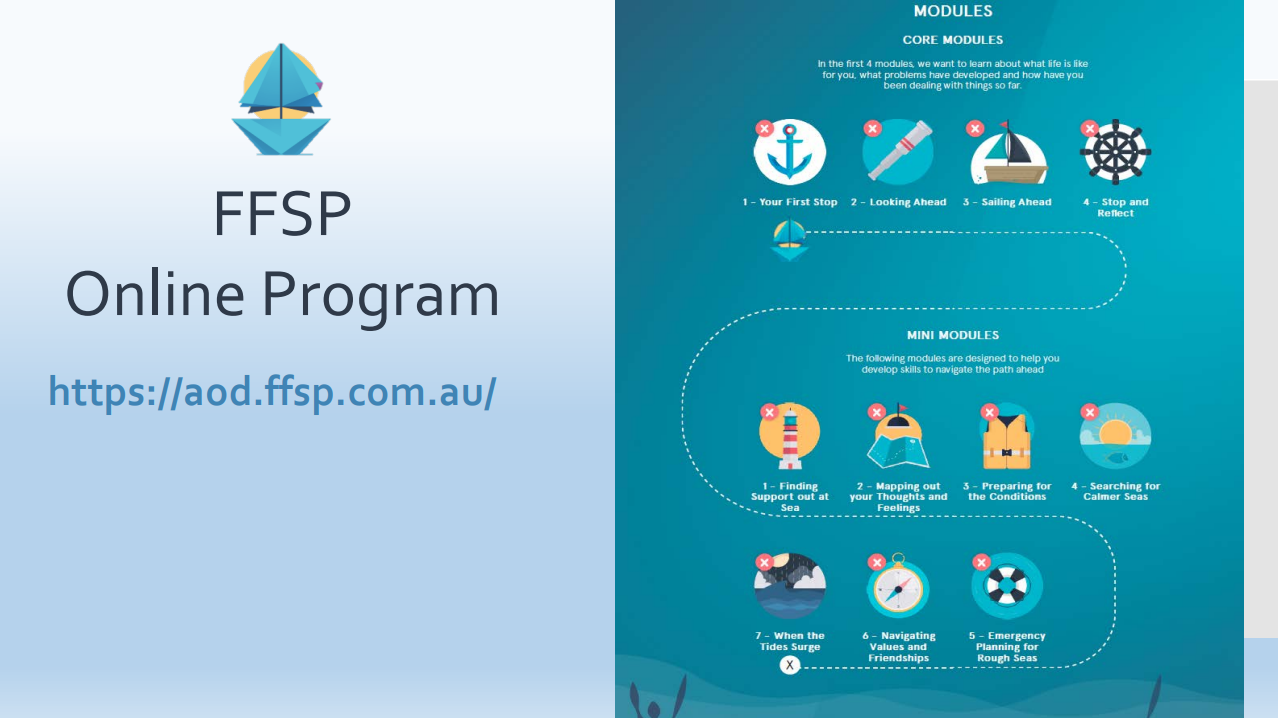


Core modules of the FFSP program


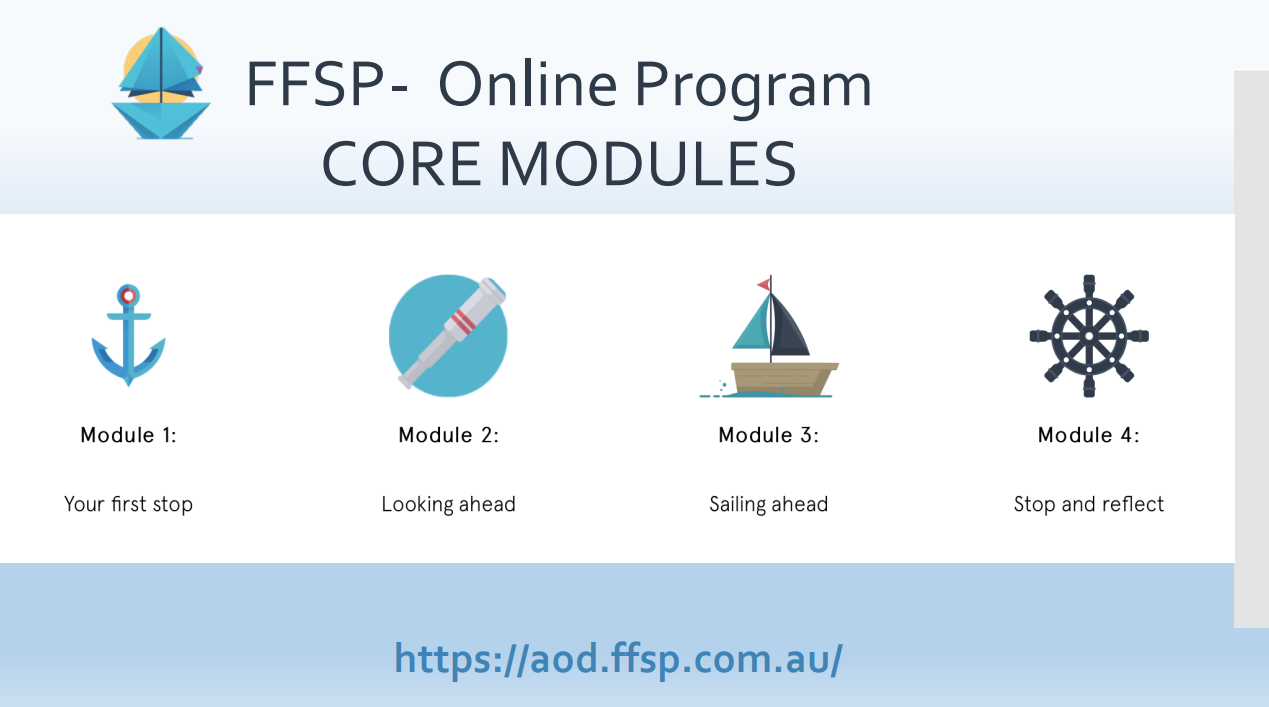


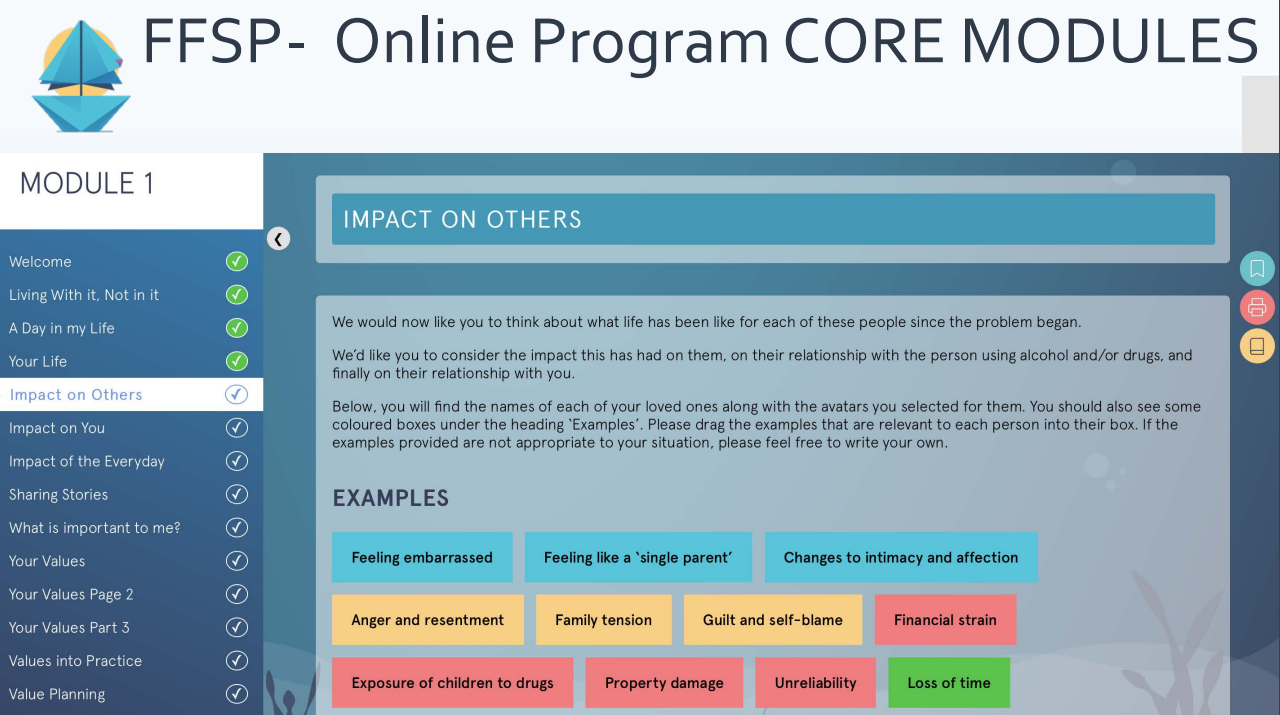


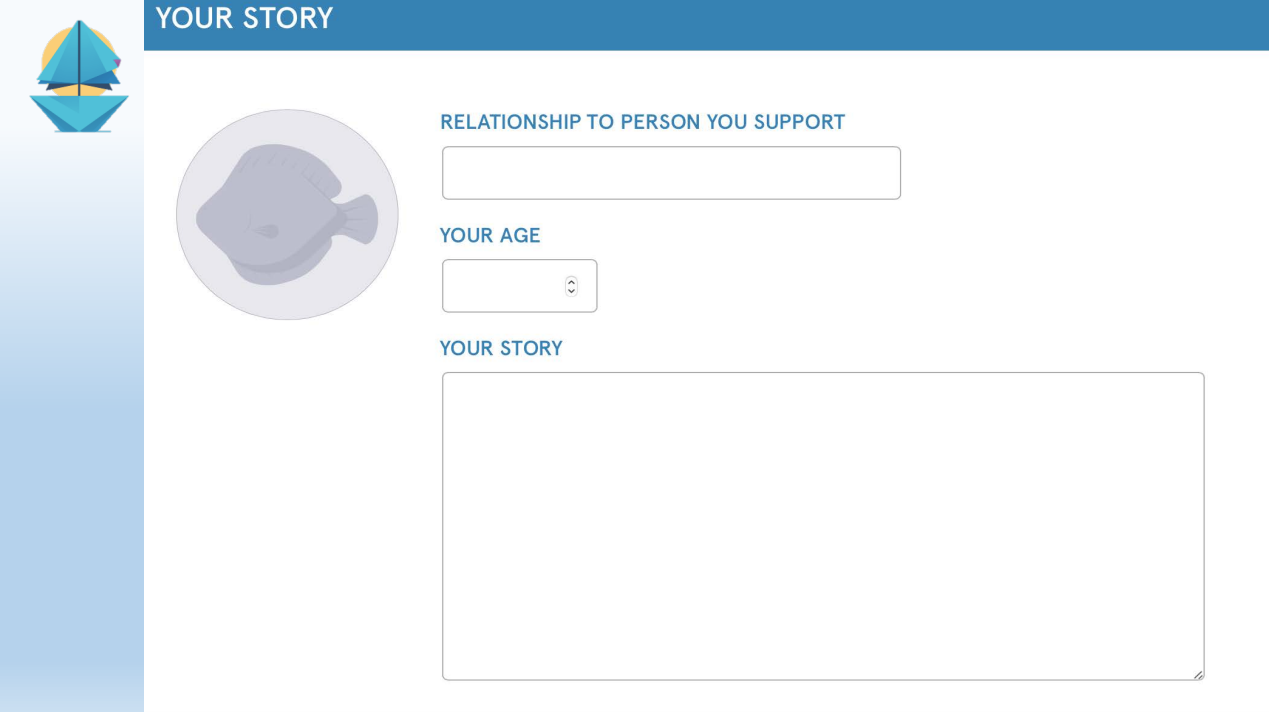


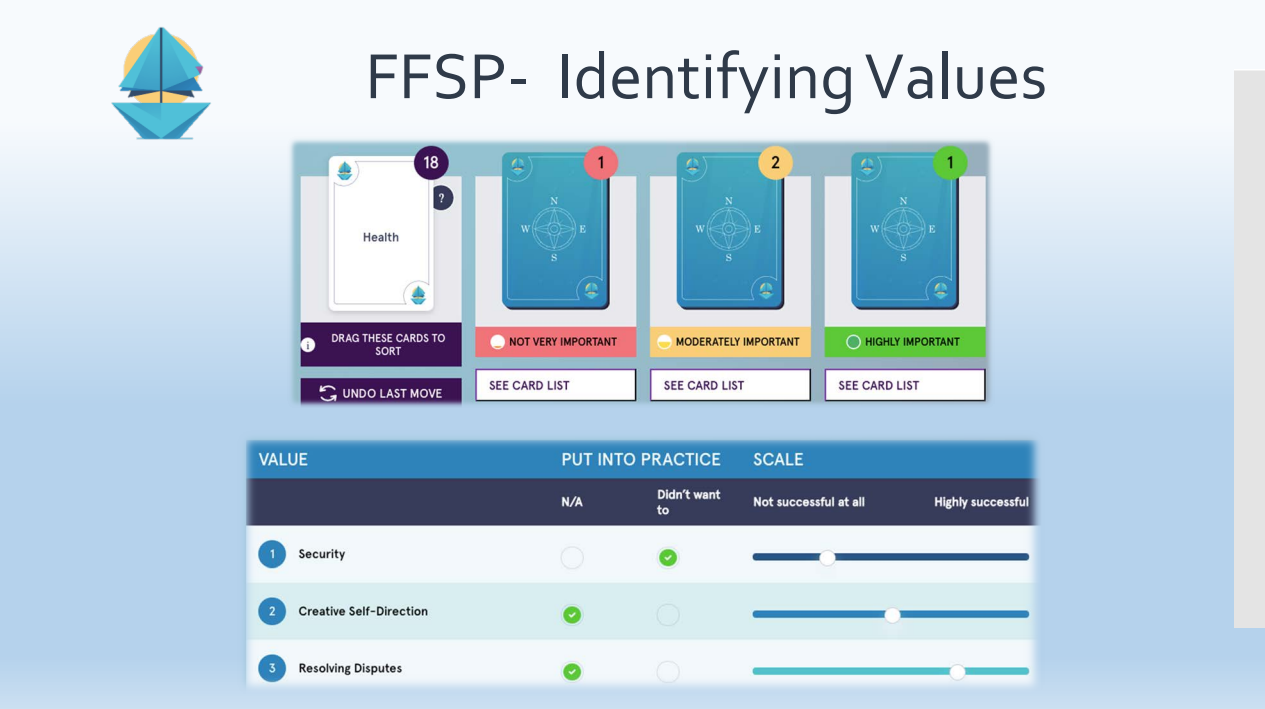


Mini modules of the FFSP program


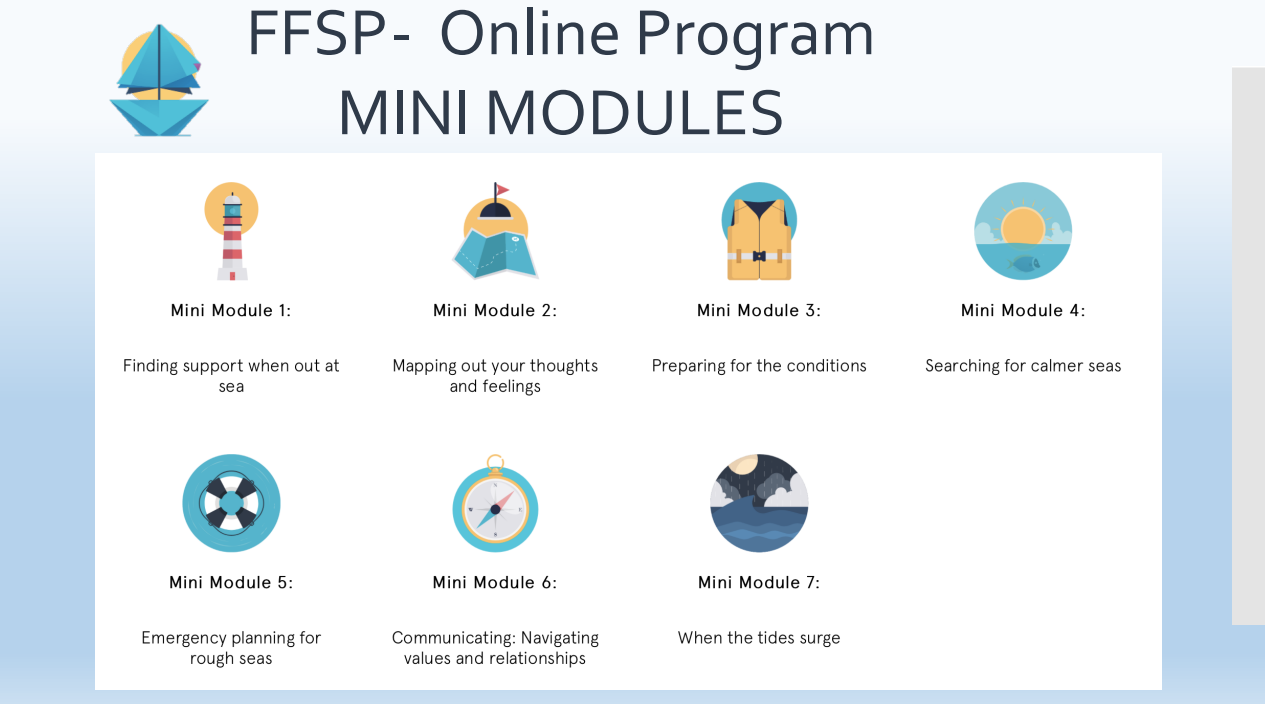


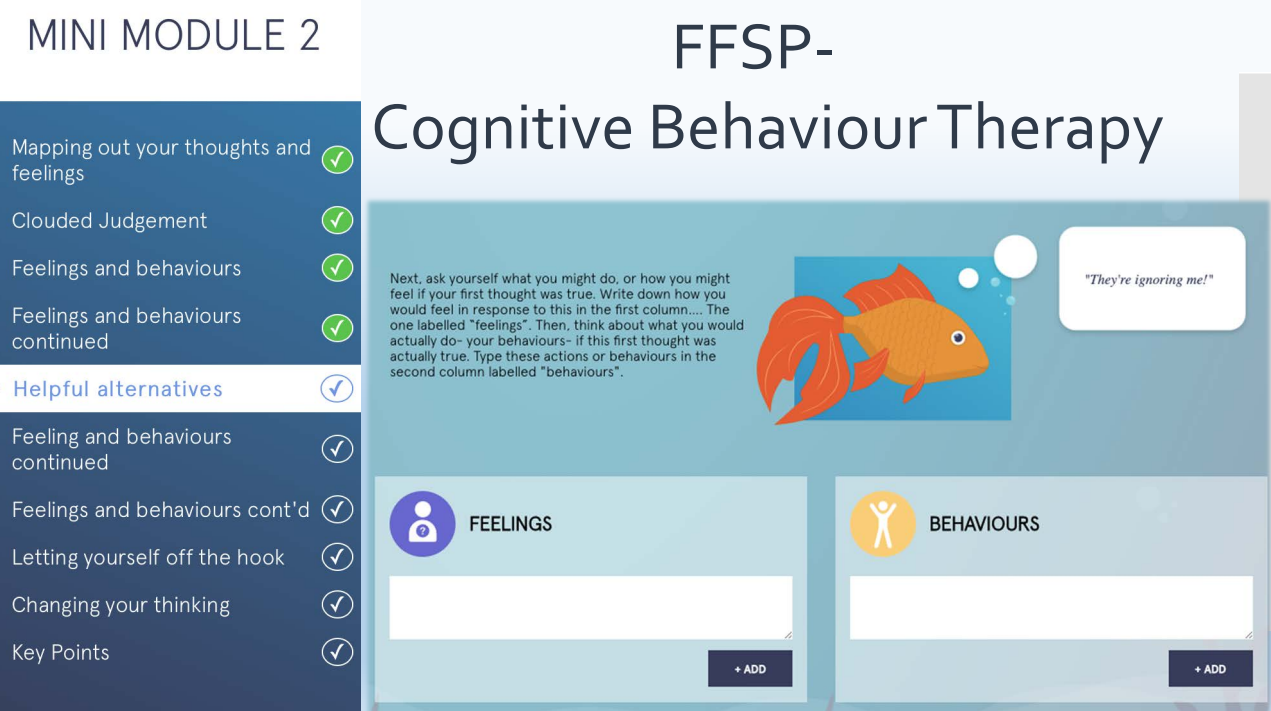

Supplement: Multimedia Appendix 1 [file formative-v9-e72425-s001.docx]
