# Supplementary material for: A Web-Based Well-Being and Resilience Intervention for Family Members and Friends Supporting a Loved One Using Alcohol and Other Drugs: Mixed Methods Pilot Study
Source: JMIR Form Res. 2025 Jul 9;9:e72425. doi: 10.2196/72425 (PMC12266297; doi:10.2196/72425)
Supplement: Multimedia Appendix 2 [file formative-v9-e72425-s002.docx]

Baseline Survey
*Informed consent, demographics, K-10, SQFM-AA*

Post-Program Survey (n = 49)
*Perceptions of the program, SUS, K-10, SQFM-AA, Help-Seeking*

Invitation to Interact with AOD FFSP (aod.ffsp.com.au) in participant’s own time/at their own pace

Follow-Up Survey (n = 32)
*Perceptions of the program, SUS, K-10, SQFM-AA, Help-Seeking*

Participants complete optional Interview (n = 5)

Additional participants complete Qualitative Phone Interview

Participants do not register an account with AOD FFSP (aod.ffsp.com.au) and do not access program (n = 32)

Participants register an account with AOD FFSP (aod.ffsp.com.au) and access program (n = 17)
