# Supplementary material for: A Web-Based Well-Being and Resilience Intervention for Family Members and Friends Supporting a Loved One Using Alcohol and Other Drugs: Mixed Methods Pilot Study
Source: JMIR Form Res. 2025 Jul 9;9:e72425. doi: 10.2196/72425 (PMC12266297; doi:10.2196/72425)
Supplement: Multimedia Appendix 3 [file formative-v9-e72425-s003.docx]

In the past 3 months, participants rated how often the following have happened as a result of their loved one’s AOD use:

| N = 131 | Never % | Once or Twice % | Sometimes % | | Often % |
| --- | --- | --- | --- | --- | --- |
| Impact |  |  |  |  | |
| Have the family’s finances been affected? | 20.6 | 11.5 | 26.0 | 42.0 | |
| Does [loved one]’s alcohol and/or other drug use get in the way of your social life? | 13.0 | 11.5 | 39.7 | 35.9 | |
| Are you worried that [loved one] has neglected their appearance or self-care? | 10.7 | 9.9 | 37.4 | 42.0 | |
| Has [loved one] picked quarrels (or fights/arguments) with you? | 9.2 | 17.6 | 32.1 | 41.2 | |
| Has [loved one] sometimes threatened you? | 45.8 | 19.8 | 18.3 | 16.0 | |
| Has [loved one] upset family or social occasions? | 13.7 | 24.4 | 32.1 | 29.8 | |
| Symptoms |  |  |  |  | |
| Worrying? | 3.8 |  | 25.2 | 71.0 | |
| Being irritable? | 6.1 | - | 61.1 | 32.8 | |
| Had thoughts you could not push out of your mind? | 13.0 | - | 41.2 | 45.8 | |
| Had parts of the body feel weak? | 34.4 | - | 48.9 | 16.8 | |
| Cannot concentrate? | 13.0 | - | 48.9 | 16.8 | |
| Awakening early and not being able to fall asleep again? | 12.2 | - | 44.3 | 43.5 | |
| Engaged Emotional Coping |  |  |  |  | |
| Started an argument with [loved one] about their alcohol and/or other drug use? | 32.8 | 32.8 | 19.8 | 14.5 | |
| Got moody or emotional with [loved one]? | 11.5 | 32.1 | 28.2 | 28.2 | |
| Watched [loved one]’s every move? | 19.8 | 25.2 | 25.2 | 29.8 | |
| Engaged Assertive Coping |  |  |  |  | |
| Sat down together with [loved one] and talked frankly about what could be done about their alcohol and/or other drug use? | 40.6 | 40.6 | 6.3 | 12.5 | |
| Made it clear that you won’t accept [loved one]’s reasons for taking alcohol and/or other drugs, or cover up for them? | 40.6 | 28.1 | 12.5 | 18.8 | |
| Made clear to [loved one] your expectations of what they should do to contribute to the family? | 21.9 | 40.6 | 12.5 | 18.8 | |
| Tolerant Inactive Coping |  |  |  |  | |
| Put yourself out for [loved one], for example by getting them to bed or by clearing up mess after they had been drinking/taking drugs? | 27.5 | 17.6 | 19.8 | 35.1 | |
| Given [loved one] money even when you thought it would be spent on drink/drugs? | 34.4 | 19.8 | 28.2 | 17.6 | |
| When things have happened as a result of [loved one]’s alcohol and/or other drugs made excuses for them, covered up for them, or taken the blame yourself? | 39.7 | 27.5 | 17.6 | 15.3 | |
| Withdrawal Coping |  |  |  |  | |
| Pursued your own interests or looked for new interests or occupation for yourself, or got more involved in a political, church, sports, or other organization? | 38.9 | 23.7 | 22.9 | 14.5 | |
| Got on with your own things or acted as if [loved one] wasn’t there? | 20.6 | 29.8 | 29.0 | 20.6 | |
| Sometimes put yourself first by looking after yourself or giving yourself treats? | 21.4 | 35.1 | 29.8 | 13.7 | |
| Helpful Informal Support |  |  |  |  | |
| Friends/relations have listened to me when I have talked about my feelings. | 10.7 | 25.2 | 33.6 | 30.5 | |
| Friends/relations have been there for me. | 9.9 | 26.7 | 30.5 | 32.8 | |
| Friends/relations have talked to me about [loved one] and listened to what I have to say. | 9.2 | 28.2 | 29.8 | 32.8 | |
| Helpful Formal Support |  |  |  |  | |
| Health/social care workers have given me helpful information about problem drinking or drug taking. | 58.0 | 17.6 | 13.0 | 11.5 | |
| Health/social care workers have made themselves available to me. | 58.8 | 22.9 | 8.4 | 9.9 | |
| I have confided in my health/social care worker about my situation. | 53.4 | 23.7 | 9.9 | 13.0 | |
| Unhelpful Informal Support |  |  |  |  | |
| Friends/relations have said things about [loved one] that I do NOT agree with. | 26.7 | 37.4 | 26.7 | 9.2 | |
| Friends/relations have said that [loved one] does NOT deserve help. | 53.4 | 19.8 | 15.3 | 11.5 | |
| Friends/relations have said nasty things about [loved one]. | 30.5 | 31.3 | 19.8 | 18.3 | |

In the past 3 months, participants rated how often the following impacts have happened as a result of their loved one’s AOD use.

In the past 3 months, participants rated how often they have experienced the following symptoms as a result of their loved one’s AOD use.

In the past 3 months, participants rated how often they have engaged with the following engaged emotional coping behaviours.

In the past 3 months, participants rated how often they have engaged with the following engaged assertive coping behaviours.

Note: Variable “Made clear to [loved one] your expectations of what they should do to contribute to the family?” selected N/A=7.6

In the past 3 months, participants rated how often they have engaged with the following tolerant inactive coping behaviours.

In the past 3 months, participants rated how often they have engaged with the following withdrawal coping behaviours.

In the past 3 months, participants rated how often they have experienced the following helpful informal support interactions.

In the past 3 months, participants rated how often they have experienced the following helpful formal support interactions.

In the past 3 months, participants rated how often they have experienced the following unhelpful formal support interactions.
