# Supplementary material for: A Web-Based Well-Being and Resilience Intervention for Family Members and Friends Supporting a Loved One Using Alcohol and Other Drugs: Mixed Methods Pilot Study
Source: JMIR Form Res. 2025 Jul 9;9:e72425. doi: 10.2196/72425 (PMC12266297; doi:10.2196/72425)
Supplement: Multimedia Appendix 4 [file formative-v9-e72425-s004.docx]

Baseline Descriptive Statistics (N = 131)

|  | Mean | Median | Min. | Max. | SD |
| --- | --- | --- | --- | --- | --- |
| K-10 | 28.42 | 28.00 | 10.00 | 50.00 | 8.62 |
| SQFM-AA |  |  |  |  |  |
| Impact | 16.86 | 17.00 | 7.00 | 24.00 | 4.09 |
| Symptoms | 13.56 | 14.00 | 6.00 | 18.00 | 2.63 |
| Engaged emotional coping | 7.54 | 7.00 | 3.00 | 12.00 | 2.54 |
| Engaged assertive coping | 7.95 |  |  |  |  |
| Tolerant inactive coping | 7.00 | 7.00 | 3.00 | 12.00 | 2.78 |
| Withdrawal coping | 6.98 | 7.00 | 3.00 | 12.00 | 2.51 |
| Helpful Informal Support | 8.56 | 9.00 | 3.00 | 12.00 | 2.67 |
| Helpful Formal Support | 5.30 | 4.00 | 3.00 | 12.00 | 2.79 |
| Unhelpful informal support | 6.29 | 6.00 | 3.00 | 12.00 | 2.55 |
| Total Family Burden | 44.96 | 44.00 | 21.00 | 65.00 | 9.46 |

Mean scores across timepoints

|  | Baseline (n = 131) | | Post Program (n = 49) | | | Follow Up (n = 32) | |
| --- | --- | --- | --- | --- | --- | --- | --- |
|  | *M* | *SD* | *M* | | *SD* | *M* | *SD* |
| K-10 | 28.42 | 8.62 | 24.43 | | 9.56 | 23.78 | 10.33 |
| SQFM-AA |  |  |  |  | |  |  |
| Impact | 16.86 | 4.09 | 15.00 | | 4.89 | 13.94 | 4.77 |
| Symptoms | 13.56 | 2.63 | 12.61 | | 2.86 | 12.69 | 3.17 |
| Engaged emotional coping | 7.54 | 2.54 | 6.75 | | 2.40 | 5.38 | 1.88 |
| Engaged assertive coping | 7.95 | 2.91 | 7.24 | | 2.75 | 6.47 | 2.58 |
| Tolerant inactive coping | 7.00 | 2.78 | 5.35 | | 2.13 | 5.13 | 2.11 |
| Withdrawal coping | 6.98 | 2.51 | 7.00 | | 2.69 | 7.47 | 2.98 |
| Helpful Informal Support | 8.56 | 2.67 | 7.65 | | 2.67 | 7.25 | 2.64 |
| Helpful Formal Support | 5.30 | 2.79 | 5.00 | | 2.18 | 4.58 | 2.29 |
| Unhelpful informal support | 6.29 | 2.55 | 5.88 | | 2.15 | 5.56 | 2.55 |
| Total Family Burden | 44.96 | 9.46 | 39.71 | | 10.05 | 37.13 | 9.35 |
